# 

**Published:** 1912-04-06

**Authors:** 


**The Modern Newspaper of Administrative Medicine and Institutional Life.**

Telegraphic Address: OSPEDALE, LONDON. Telephone Number: 2734 GERRARD.

No. 1343, Vol. LII. | Saturday, April 6th, 1912. | PRICE ONE PENNY

